# Downregulation of RNF128 activates Wnt/β-catenin signaling to induce cellular EMT and stemness via CD44 and CTTN ubiquitination in melanoma

**DOI:** 10.1186/s13045-019-0711-z

**Published:** 2019-03-04

**Authors:** Chuan-Yuan Wei, Meng-Xuan Zhu, Yan-Wen Yang, Peng-Fei Zhang, Xuan Yang, Rui Peng, Chao Gao, Jia-Cheng Lu, Lu Wang, Xin-Yi Deng, Nan-Hang Lu, Fa-Zhi Qi, Jian-Ying Gu

**Affiliations:** 1 Department of Plastic Surgery, Zhongshan Hospital, Fudan University, Shanghai, 200032 People’s Republic of China; 2 Department of Liver Surgery and Transplantation, Liver Cancer Institute and Zhongshan Hospital, and Key Laboratory of Carcinogenesis and Cancer Invasion (Ministry of Education), Fudan University, Shanghai, People’s Republic of China; 3 Liver Cancer Institute, Zhongshan Hospital, Fudan University, Shanghai, People’s Republic of China; 4 Department of Oncology, Shanghai East Hospital, Tongji University School of Medicine, Shanghai, 200032 People’s Republic of China

**Keywords:** RNF128, Melanoma, EMT, Prognosis, Wnt signaling

## Abstract

**Background:**

Ring finger proteins (RNFs) were involved in carcinogenesis. Here, we aimed to explore the detailed mechanism of RNF128 in the progression of melanoma.

**Methods:**

We reanalyzed several gene expression profiles from the Gene Expression Omnibus (GEO) database and obtained the overlapped differential expressed RNF genes. Among them, RNF128 was selected to further explore its expression, the biological significance, and the underlying molecular mechanism, as well as the clinical relevance in melanoma patients.

**Results:**

RNF128 was found to be significantly downregulated in the selected datasets, which was further verified in our melanoma tissues. Moreover, RNF128 downregulation was shown to correlate with the malignant phenotype of melanoma, and further functional assays demonstrated that low levels of RNF128 promoted melanoma progression via inducing cell epithelial-mesenchymal transition (EMT) and the acquisition of stemness. Mechanistically, RNF128 interference activated the Wnt pathway via simultaneously ubiquitinating CD44/cortactin (CTTN), resulting in CD44 and c-Myc transcription, thus revealed that RNF128 participated in a positive feedback of the Wnt pathway-CD44 loop. Clinically, we found that patients expressing low RNF128 and high CD44/CTTN levels had a poor prognosis.

**Conclusion:**

Downregulated RNF128 activates Wnt signaling to induce cellular EMT and stemness by ubiquitinating and degrading CD44/CTTN, and RNF128 is a reliable diagnostic and prognostic biomarker, and a deeper understanding of RNF128 may contribute to the treatment of melanoma.

**Electronic supplementary material:**

The online version of this article (10.1186/s13045-019-0711-z) contains supplementary material, which is available to authorized users.

## Introduction

The incidence of melanoma is growing globally, with approximately 280,000 new cases diagnosed every year [1]. Although accounts for a small proportion of cutaneous tumors, melanoma is responsible for the greatest number of skin cancer-related deaths and causes nearly 55,500 deaths annually [2], which is mainly related to the distant metastatic spread of melanoma [3]. Now, the initiation and development of melanoma were identified to be caused by dysfunctions of oncogenic and tumor suppressor pathway, and different biomarkers have been identified to be highly significant and relevant in the context of melanoma, including BRAF, NRAS, and C-KIT [4–6]. Of these, 50% of melanomas harbor mutations in BRAF, mainly at codon 600, which results in the continuous activation of the MAPK pathway [7]. Although targeted drugs, such as vemurafenib and dabrafenib, have improved the survival of melanoma patients, the prognosis remains dismal [2]. Thus, it is critical to discover new biomarkers that drive the initiation and progression of melanoma, which may help to develop new targets for diagnosis and treatment.

Ubiquitination plays an essential role in protein posttranslational modification and is strongly linked to different biological and pathological processes in eukaryotes [8]. Ubiquitin-protein enzymes (E3s) are of particular concern in this process, as they not only transfer activated ubiquitin from ubiquitin-conjugating enzymes to protein substrates but also confer substrate specificity [9]. The RING finger protein family, a complex set of E3s that contain an RNF domain, was recently demonstrated to play crucial roles in tumorigenesis and tumor progression [10]. For example, RNF13 is an ER/Golgi membrane-associated E3, and overexpressed RNF13 increases the invasive potential and gelatinolytic activity of pancreatic cancer by increasing matrix metalloproteinase-9 (MMP9) activity [11]. Another example is RNF183, which contributes to the progression from inflammation to malignancy by activating the NF-κB-IL-8 axis in colorectal cancer [12]. Identifying more cancer-related RNF family members will help us to better understand the mechanisms of tumor progression and develop new therapeutic strategies.

In the current study, we reanalyzed the available gene expression profiles from the GEO database and revealed that RNF128 was consistently downregulated in the selected datasets. Here, we also demonstrated that low level of RNF128 was closely related to Breslow depth, Clark level, distant metastasis, and TNM stage of melanoma. Moreover, RNF128 interference promoted cellular EMT and the acquisition of stemness by activating the Wnt pathway via ubiquitinating and degrading the CD44 and CTTN proteins, resulting in the transcription of CD44 and c-Myc, which indicated that RNF128 participated in a positive feedback of the Wnt signaling-CD44 loop. Thus, our study indicates that low level of RNF128 is a promoter of melanoma, and a deeper understanding of RNF128 may contribute to the diagnostic, prognostic, and therapeutic strategies.

## Materials and methods

### Data availability

To identify relevant RNF family members that are critical in the pathogenesis of melanoma, we performed data mining on the GEO database (https://www.ncbi.nlm.nih.gov/gds/). GSE3189 was based on the GPL96 platform (HG-U133A, Affymetrix Human Genome U133A Array), and including 45 melanoma and 7 normal tissues, and GSE7553 was based on the GPL570 platform (HG-U133_ plus 2, Affymetrix Human Genome U133A Plus 2.0 Array), and including 14 melanoma and 4 normal tissues. GEO2R (https://www.ncbi.nlm.nih.gov/geo/geo2r/) was used to calculate the adjusted *p* values and logFC values among different groups. GSEA was performed using GSEA 2.2.1 (http://www.broadinstitute.org/gsea).

### Patients and follow-up

A total of 138 paraffin-embedded melanoma and matched peritumoral tissues and an additional 58 melanoma tissues were collected to construct the tissue microarray (TMA). Thirty pairs of frozen melanoma and matched nontumor tissues were randomly selected and analyzed by quantitative real-time polymerase chain reaction (qRT-PCR) and western blot. All patients underwent curative resection verified by pathological examination at Zhongshan Hospital of Fudan University (Shanghai*,* China). Clinicopathological information was collected from 1 January 2008 to 31 December 2017. The Ethics Committee of the Zhongshan Hospital Biomedical Research Department provided ethical approval, and informed consent for collecting and preserving samples and details was obtained from each patient.

### Cell culture and transfection

The human melanoma cell lines A2058, A375, A875, MV3, M14, and Sk-mel-28 were purchased from the cell bank of the Chinese Academy of Sciences (Shanghai, China). These cells were cultured in DMEM or RPMI-1640 medium (HyClone, USA) with 10% fetal bovine serum (Invitrogen, USA), penicillin (100 IU/ml), and streptomycin sulfate (100 μg/ml) at 37 °C in a thermostatic incubator containing 5% CO_2_. pLVX-shRNA-eGFP-PGK-Puro and CMV-H_RNF128-eGFP-3flag-PGK-Puro lentiviral vectors were purchased from Genomeditech (Shanghai, China). The pLVX-shRNA-eGFP-PGK-Puro lentiviral vectors were transfected into M14 cells, and the pGMLV-SC5-Puromycin vectors were used as negative controls. The CMV-H_RNF128-eGFP-3flag-PGK-Puro lentiviral vectors were transfected into A2058 cells. siRNAs against Snail, CD44, CTTN, and β-catenin were designed and synthesized by Genomeditech (Shanghai, China). The target sequences were as follows: siSnail, GCGTGGGTTTTTGTATCCA; siCD44, CTGAAATTAGGGCCCAATT; siCTTN, CCTTAAGGAGAAGGAACTT; and siβ-catenin, TGGTTGCCTTGCTCAACAA. The siRNA was transfected using Lipofectamine™ 2000 (Thermo Fisher Scientific, USA) according to the manufacturer’s protocols. The efficiency of silencing was confirmed by western blot and qRT-PCR after 72 h of transfection.

### TMA construction and IHC staining

The construction of TMA and immunohistochemistry (IHC) staining were performed as described previously [13, 14]. Briefly, the slide was deparaffinized, rehydrated, subjected to antigen retrieval, and incubated in 0.3% H_2_O_2_. Subsequently, the sections were incubated with the primary antibody (listed in Additional file 1: Table S2) at 4 °C overnight and then stained with horseradish peroxidase-labeled IgG (Gene Tech, China). Then, the section was stained with diaminobenzidine, counterstained with hematoxylin, dehydrated in ethanol, cleared in xylene, and cover-slipped. The density of positive staining was measured as previously described [13]. Briefly, images of 4 representative fields were captured under high-power magnification (× 200), and identical settings were used for all of the images. The integrated absorbance and area of the images were counted by Image-Pro Plus v6.0 software (Media Cybernetics, Inc., Bethesda, MD, USA), and uniform settings were applied for all slides. The average density was calculated as the product of the integrated absorbance/total area, and the sections were classified as either high or low expression.

### qRT-PCR and western blot analysis

Total RNA was extracted from both the tissues and cultured cells using TRIzol reagent (Invitrogen, USA) and reverse-transcribed to cDNA with a PrimeScript RT Reagent Kit (Takara, Japan) according to the manufacturer’s instructions. The cycling conditions were 94 °C for 15 s, 60 °C for 30 s, and 72 °C for 30 s. Each reaction was performed in triplicate. The primer sequences for qRT-PCR are shown in Additional file 2: Table S1. Western blot was performed as described in a previous study [15], and all the primary antibodies are listed in Additional file 1: Table S2.

### Matrigel invasion and wound-healing migration

For invasion assays, cells were incubated using 24-well transwell plates (8-μm pore size, Corning, NY, USA). One million cells suspended in serum-free medium were plated in the upper chambers with Matrigel (BD Biosciences, USA), and 0.6 ml of DMEM or RPMI-1640 medium with 10% FBS was added to the lower chamber. After incubation for a suitable amount of time, the cells were fixed in 4% paraformaldehyde, stained by crystal violet, and counted under a microscope. For wound-healing migration assays, the cell monolayers were mechanically disrupted using a sterile 200-μl pipette tip to generate a linear wound. The average distance migrated by the cells was measured using a microscope calibrated with an ocular micrometer at a suitable time.

### Colony formation, sphere formation, and CCK-8 assays

The colony formation assay was performed as described in a previous study [16]. Briefly, cells were seeded in a 6-cm culture dish (1000 cells), and the culture medium was refreshed every 3 days for 2 weeks. After that, the cells were washed with PBS, fixed with 4% paraformaldehyde, and stained with 0.4% crystal violet for 15 min. The number of colonies containing > 10 cells was counted manually and averaged over duplicate wells. For sphere formation assay, cells were plated in ultralow attachment 6-well plate (Corning Inc., USA) at the density of 1000 cells per well in a 2 ml of serum-free DMEM/F12 basal medium supplemented with l-glutamine (2 mM), 20 ng/ml human epidermal growth factor, 20 ng/ml human fibroblast growth factor-2, and B-27 supplement (1:50) at 37 °C for 2 weeks. After that, the diameters of each cell sphere were measured, and the numbers of the sphere with a diameter > 100 μm were counted as primary spheres. Cell proliferation was detected by the Cell Counting Kit-8 (CCK-8, Yeasen, Shanghai, China) and performed as described in a previous study [17]. Briefly, cells were inoculated into 96-well plates (1000 cells per well). Then, 10 μl of CCK-8 reagent (Yeasen, Shanghai, China) was added to the wells after the first, second, third, and fourth days. The plates were incubated for 2 h, and the absorbance was determined at 490 nm.

### Flow cytometric and immunofluorescence assays

Flow cytometric analysis was performed as in a previous study [18] and was used to determine the percentage of positively stained cells. A total of 10^5^ cells were collected in the tube and stained with Annexin V-APC/7-ADD (Yeasen, Shanghai, China) or PE-CD133 (BioLegend, CA, USA). Positively stained cells were quantified by flow cytometry (Becton Dickinson) and analyzed by FlowJo-V10 software. Immunofluorescence was used to detect the location and expression of target proteins, as described previously [19]. Briefly, after being fixed with 4% paraformaldehyde, incubated in 0.3% Triton X-100 and blocked with 5% FBS, cells were incubated with primary antibodies at 4 °C overnight, followed by incubation with the appropriate secondary antibody (Yeasen, Shanghai, China). The nuclei were counterstained with 4, 6-diamidino-2-phenylindole (DAPI, Yeasen, Shanghai, China). The intensity of fluorescence was detected by confocal laser scanning microscopy (LSM510; Zeiss, Germany).

### Metastasis in vivo

Xenograft experiments in nude mice were approved by the Animal Experimentation Ethics Committee of Zhongshan Hospital, Fudan University. Male BALB/c nude mice aged 4–6 weeks were maintained according to the stated guidelines of the 3 Rs (replacement, reduction, and refinement). All mice were randomized, and the investigators were blinded to the group assignment. We resuspended 10^6^ cells (per mouse) in 100 μl of PBS and injected them into the lateral tail vein. The mice were sacrificed after 30 days; the lungs were resected, embedded in paraffin, and stained with hematoxylin and eosin (H&E); and lung metastases were counted.

### IP assay and MS

Cells were harvested in lysis buffer supplemented with a protease inhibitor. After removing the insoluble material by 12,000×*g* centrifugation, the protein concentration was determined using the Bradford method (Bio-Rad, Hercules, CA, USA). Precleared lysates with equivalent amounts of protein were incubated with a primary antibody overnight at 4 °C. Then, protein A- and G-Sepharose beads (Pierce Biotechnology, Rockford, IL, USA) were added to the immunoprecipitation (IP) mixture for 2 h. The precipitates were washed with lysis buffer three times, resuspended in SDS-PAGE sample buffer, boiled, and loaded onto 8% gradient gels. Subsequent western blots were probed with the target antibody and detected by enhanced chemiluminescence (Millipore, Darmstadt, Germany).

Mass spectrometry (MS) assay was performed to detect the potential interacting proteins, as described previously [20]. Briefly, the immunoprecipitates described above were resolved on 10% gradient gels, and the protein bands of interest were visualized by Coomassie blue staining and used for MS analysis. MS was operated in reflection mode with an m/z range of 400 to 2000 and a 19 kV accelerating voltage. All MS data were identified using SEQUEST (v.28, Thermo Electron) against the Human International Protein Index database.

### Ubiquitination assay and CHX chase assay

M14-shNC and M14-shRNF128 cells were transiently transfected with hemagglutinin (HA)-tagged ubiquitin vectors. Forty-eight hours later, the cells were washed with PBS and incubated with 10 μM MG132 (Selleck, Texas, USA) for 8 h. Then, the cells were lysed and subjected to immunoprecipitation with anti-CD44 and anti-CTTN antibodies. Ubiquitination of substrates was analyzed by SDS-PAGE after blotting with an anti-HA antibody. The half-life of CD44 and CTTN was determined by a CHX chase assay. M14-shNC and M14-shRNF128 cells were treated with cycloheximide (CHX) (100 mg/mL) for the indicated times, and western blot was performed to detect the expression of CD44 and CTTN.

### TOP/FOP luciferase reporter assay

The TOPFlash/FOPFlash luciferase reporter assay was performed using the Luciferase Assay System (Promega) according to the manufacturer’s instructions. Briefly, cells were co-transfected with the Wnt/β-catenin signal pathway reporter TOPFlash/FOPFlash. After 24 h of plasmids transfection, cells were lysed and luciferase activity was measured using the Dual-Luciferase Reporter Assay Kit (Promega). The luciferase activities of Firefly and Renilla were determined using a luminometer.

### Statistical analysis

All in vitro experiments were repeated at least three times. The data were analyzed using IBM SPSS Statistics 20 (IBM Corp., USA), and the values are presented as the mean ± standard deviation (SD). Student’s *t* test or Tukey’s multiple comparisons test was used for comparisons between two groups, and one-way ANOVA was used for multiple group comparisons. Correlations between the two groups were detected by analysis of Pearson’s correlation coefficient. OS and recurrence rates were analyzed using the Kaplan-Meier method and the log-rank test. Independent prognostic factors were analyzed by Cox’s proportional hazards regression model. All statistical tests were two-sided, and differences were considered statistically significant at *p* < 0.05.

## Results

### RNF128 is downregulated in melanoma tissues

**Fig. 1:**
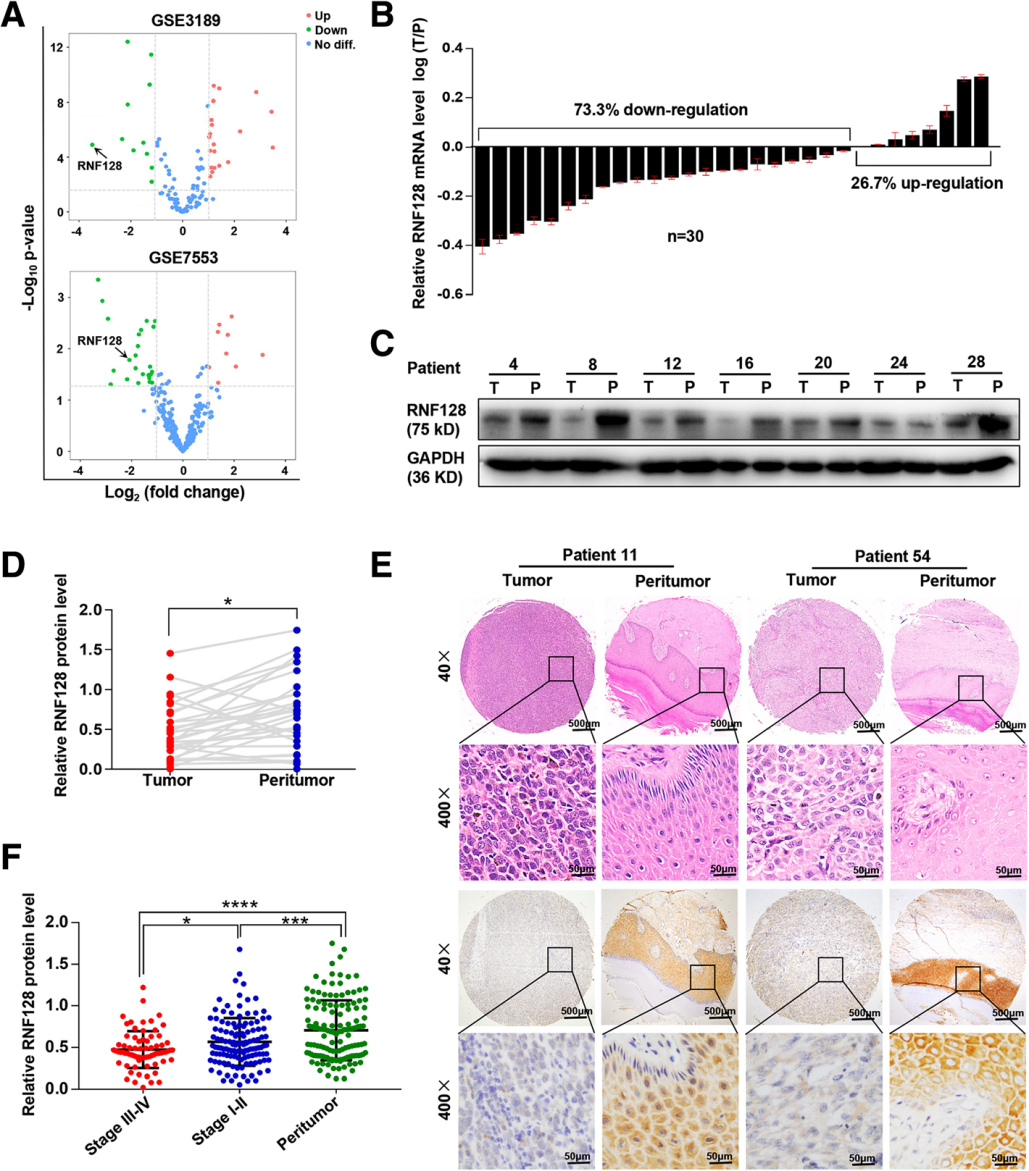
RNF128 is downregulated in melanoma tissues. **a** Volcano plots showing the differentially expressed genes among RNF family members in melanoma tissues compared with normal tissues from GSE3189 and GSE7553. The cut-off was |Log_2_FC| ≥ 1 and *p* value < 0.01. **b** The RNF128 mRNA levels in 30 pairs of melanoma and matched peritumorous tissues, shown as log (T/P). **c**, **d** The RNF128 protein levels in 30 pairs of melanoma and matched peritumorous tissues (**d**); representative bands are shown (**c**). **e** Representative images of TMA stained with H&E and IHC for anti-RNF128. **f** The RNF128 expression levels in stage III–IV and stage I–II melanoma tissues and peritumoral tissues analyzed by average densitometry. ^*^p < 0.05, ^***^p < 0.001, ^****^p < 0.0001. T, tumor; P, peritumor

To reveal dysregulated RNF family members in melanoma, we analyzed the available microarray data (GSE3189 and GSE7553, Fig. 1a), and 10 RNF genes were obtained (Additional file 3: Figure S1). Among them, RNF128 was consistently downregulated in both datasets and was chosen for the following research. We found that RNF128 was remarkably lower in melanoma tissues than in peritumoral tissues at both the mRNA (Fig. 1b) and protein levels (*p* = 0.0276, Fig. 1c, d). Additionally, immunohistochemistry revealed that the RNF128 protein was mainly distributed in the cytoplasm of melanoma cells (Fig. 1e). Through quantification analysis, we further showed that the expression of RNF128 was noticeably downregulated in melanoma compared with that in peritumoral tissues, especially in stage III–IV melanoma (*p*_(III–IV vs I–II)_ = 0.0175, *p*_(I–IIvsP)_ = 0.0006, *p*_(III–IVvsP)_ = < 0.0001, Fig. 1f). Thus, RNF128 is downregulated in melanoma.

### RNF128 downregulation promotes melanoma progression both in vitro and in vivo assays

**Fig. 2:**
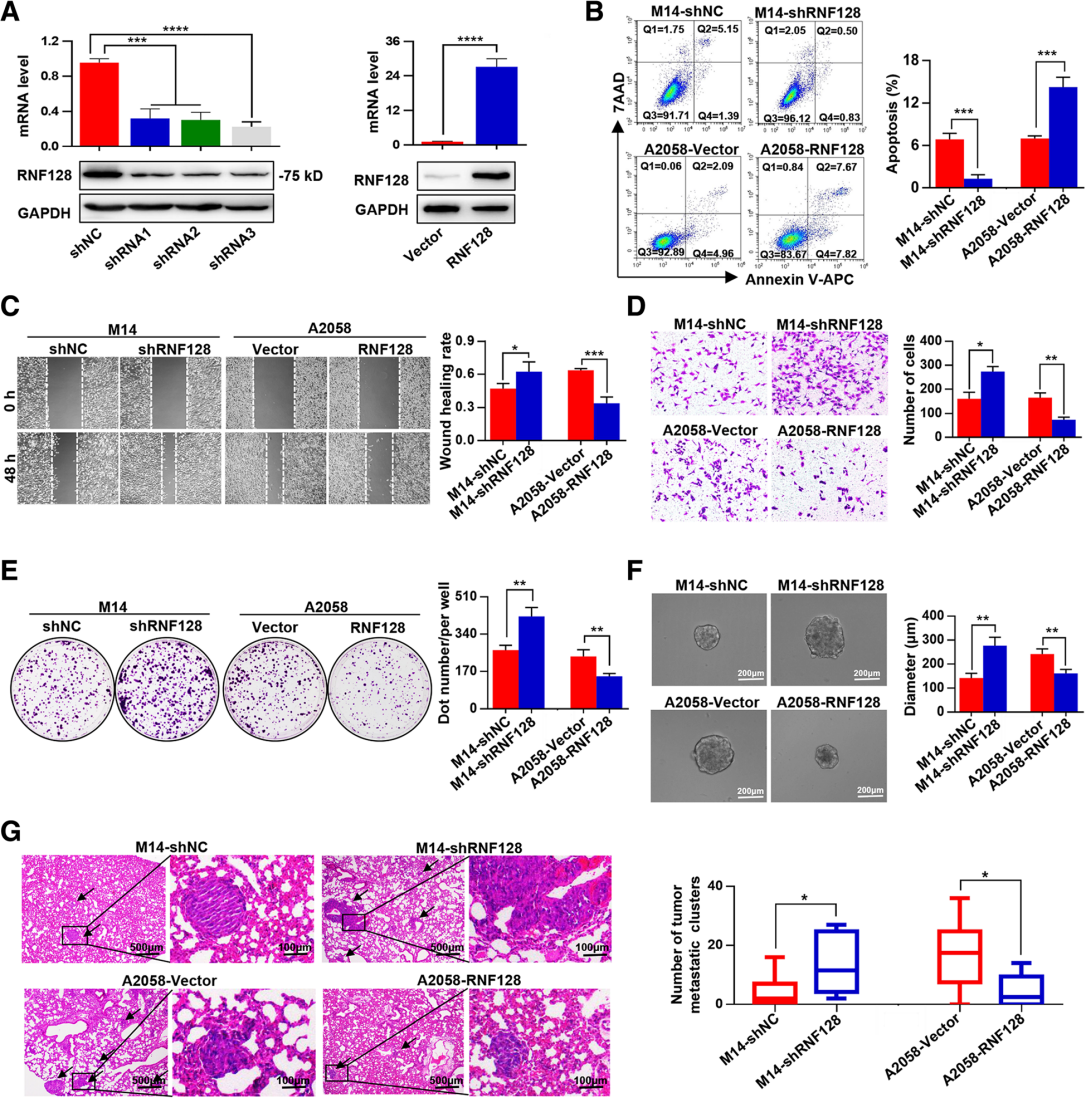
RNF128 downregulation promotes melanoma progression both in vitro and in vivo. **a** The efficiencies of RNF128 inhibition and overexpression were examined by western blot and qRT-PCR. **b** The effects of RNF128 inhibition and overexpression on cellular apoptosis were detected by flow cytometric analyses. **c**, **d** The effects of RNF128 knockdown or overexpression on cellular invasion and migration were measured by wound-healing migration assays (**c**) and Matrigel invasion assays (**d**). **e** The effects of RNF128 inhibition and overexpression on colony-forming ability were detected by colony formation assays. **f** The effects of RNF128 knockdown or overexpression on sphere-forming ability were detected by sphere-forming assays. **g** The effects of RNF128 inhibition and overexpression on lung metastasis were investigated in vivo assays, and the number of metastases was examined by H&E staining. ^*^p < 0.05, ^**^p < 0.01, ^***^p < 0.001

Here, we further determined the expression of RNF128 in six human melanoma cell lines (Additional file 4: Figure S2). Of these cells, A2058 cells with the lowest expression of RNF128 were transfected with a RNF128 cDNA vector, and M14 cells with the highest expression level of RNF128 were transfected with a RNF128-shRNA lentivirus. After the validation of transfection efficiency (Fig. 2a), shRNA3 was selected for the subsequent experiments (termed shRNF128). We found that RNF128 knockdown enhanced the invasion, migration, and apoptosis resistance of M14 cells, and conversely, RNF128 overexpression remarkably inhibited invasion, migration, and apoptosis resistance in A2058 cells (Fig. 2b–d, Additional file 5: Figure S3A). Interestingly, we found that low levels of RNF128 increased, and high level of RNF128 decreased the clone-forming and sphere-forming ability of melanoma cells (Fig. 2e, f), with no influence on their proliferation (Additional file 5: Figure S3B). Furthermore, we found that RNF128 knockdown promoted, while RNF128 overexpression inhibited lung metastasis of melanoma cells through in vivo assays (Fig. 2g). Collectively, these results show that RNF128 downregulation promotes melanoma progression.

### Downregulation of RNF128 induces EMT and stemness in melanoma cells

**Fig. 3:**
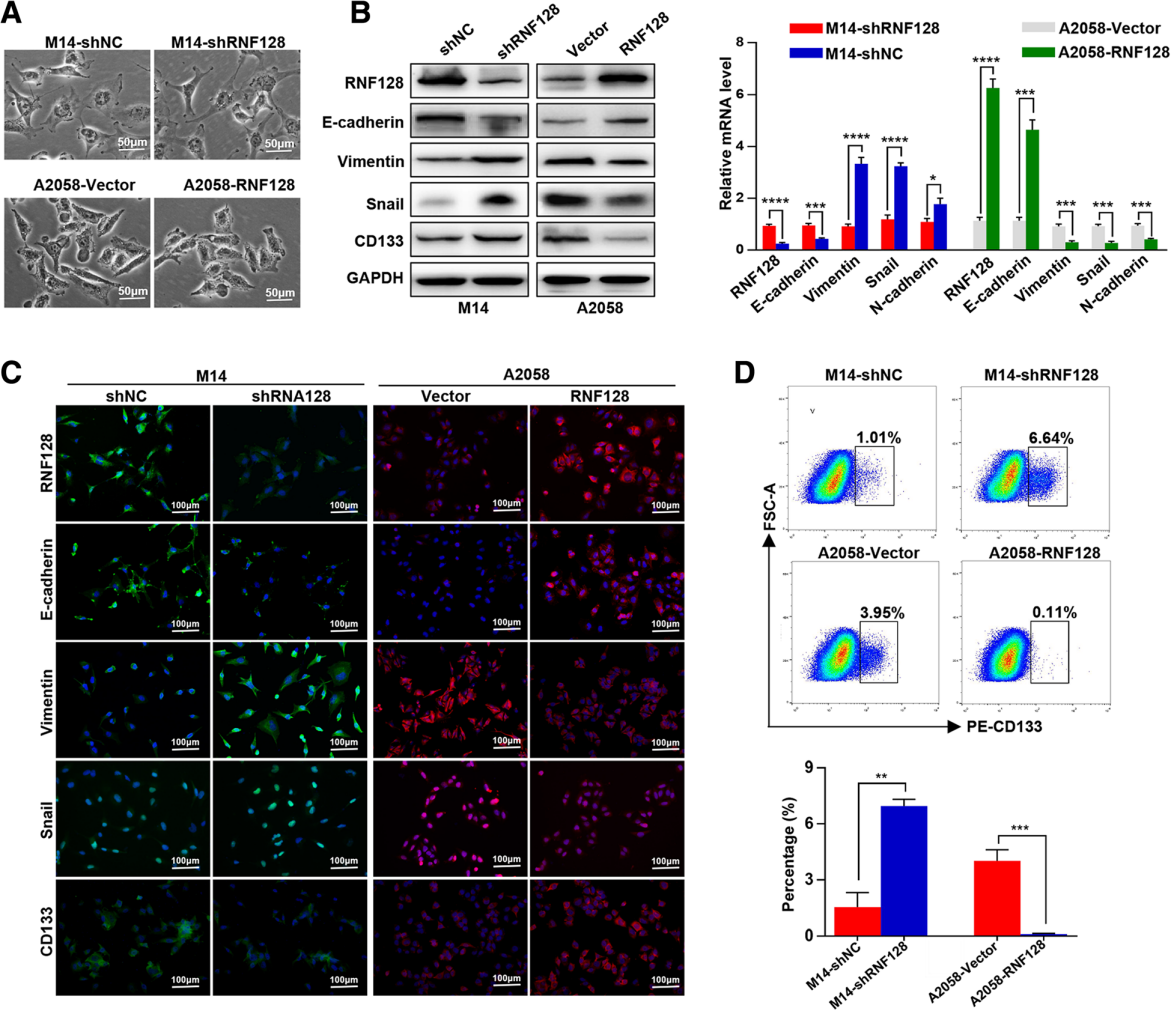
Downregulation of RNF128 induces EMT and stemness in melanoma cells. **a** The cellular morphologies in M14-shNC/M14-shRNF128 and A2058-Vector/A2058-RNF128 cells. **b** Western blot (left) and qRT-PCR (right) analyses were used to detect the expression of EMT markers (E-cadherin, vimentin, and Snail) and stemness markers (CD133) in M14-shNC/M14-shRNF128 and A2058-Vector/A2058-RNF128 cells. **c** The expression and location of RNF128, E-cadherin, vimentin, Snail, and CD133 markers in M14-shNC/M14-shRNF128 and A2058-Vector/A2058-RNF128 cells were analyzed by immunofluorescence staining. **d** Flow cytometric analyses were used to detect the expression of CD133 in the indicated cells. ^*^p < 0.05, ^**^p < 0.01, ^***^p < 0.001, ^****^p < 0.0001

We further examined RNF128 expression on melanoma cell EMT and stemness. We observed that M14-shNC and A2058-RNF128 cells took on the typical cobblestone-like appearance of normal epithelial cells, while M14-shRNF128 and A2058-Vector cells presented a spindle-like, fibroblastic morphology (Fig. 3a). Then, western blot showed that M14-shRNF128 and A2058-Vector cells expressed a low level of E-cadherin and high levels of vimentin, Snail (EMT markers), and CD133 (stemness marker) compared with those in M14-shNC and A2058-RNF128 cells through western blot and qRT-PCR (Fig. 3b). Furthermore, the expression of E-cadherin, vimentin, Snail, and CD133 were detected by immunofluorescence and flow cytometry analysis, and coincident with the results of western blot and qRT-PCR (Fig. 3c, d). Thus, we revealed that RNF128 downregulation promotes cell EMT and stemness.

### RNF128 interacts directly with CD44 and CTTN and degrades them via ubiquitination

**Fig. 4:**
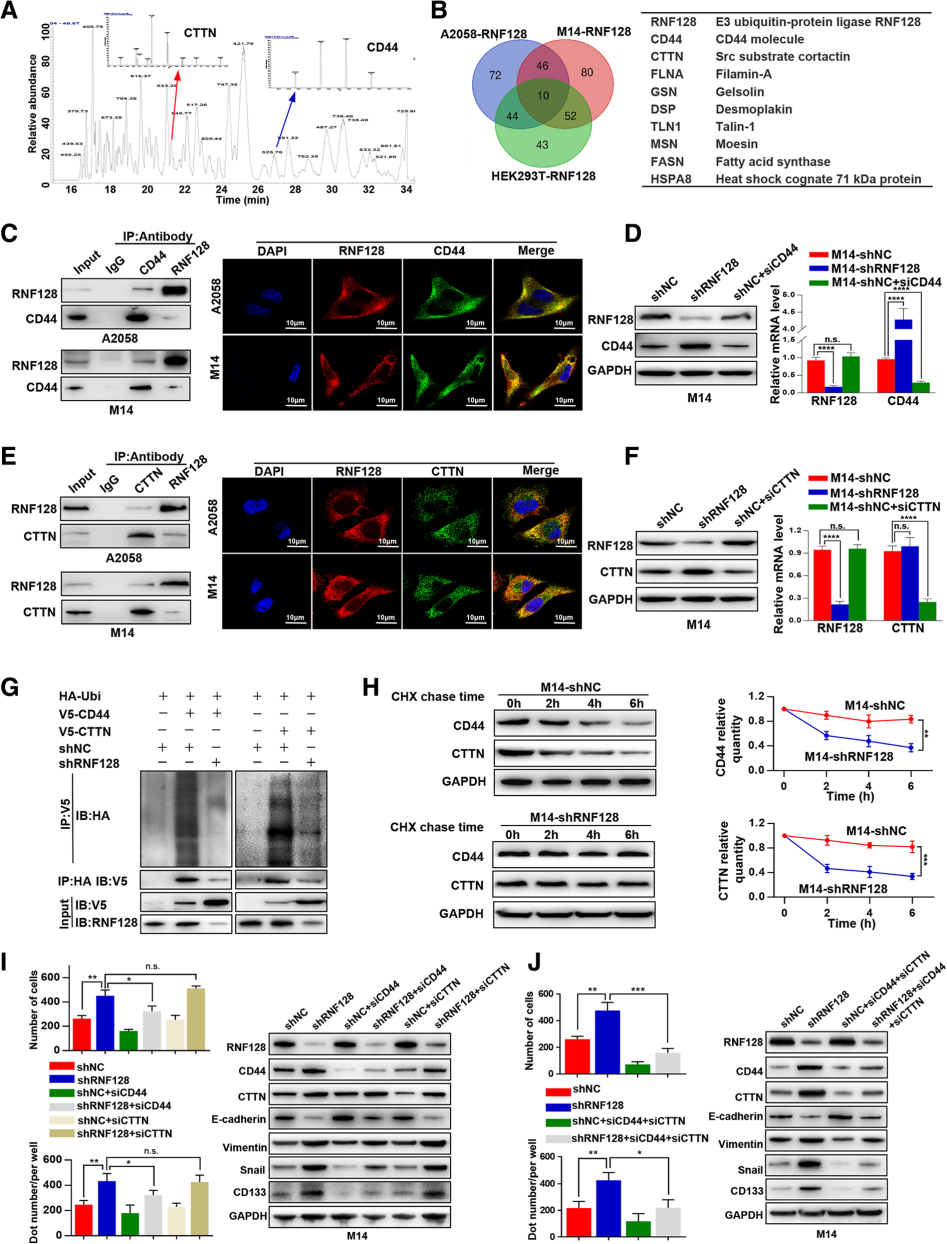
Low level of RNF128 regulates melanoma cellular EMT and stemness via simultaneously protecting CD44 and CTTN from degradation. **a** Identification of the binding partners of RNF128 by a combination of Co-IP and MS. **b** Venn diagram showing the number of binding partners of RNF128 in A2058-RNF128, M14-RNF128, and HEK-293 T-RNF128 (left), and 10 overlapping proteins are presented in the table (right). **c** Co-IP (left) and immunofluorescence assays (right) were used to detect the direct interaction of RNF128 and CD44. **d** Western blot and qRT-PCR were used to detect the expression of RNF128 and CD44 in the indicated cells. Low level of RNF128 upregulated the expression of CD44 both in protein and mRNA level, while interfered CD44 did not influence the RNF128 expression. **e** Co-IP (left) and immunofluorescence assays (right) were used to detect the direct interaction of RNF128 and CD44. **f** Western blot and qRT-PCR were used to detect the expression of RNF128 and CTTN in the indicated cells. Low level of RNF128 upregulated the expression of CTTN in protein level, while interfered CTTN did not influence the RNF128 expression. **g** Impact of M14-shNC and M14-shRNF128 cells on CD44 or CTTN ubiquitination. V5-CD44 or V5-CTTN and HA-Ubi were co-transfected into M14-shNC and M14-shRNF128 cells, and the cell lysates were immunoprecipitated with the indicated antibodies and analyzed by western blot. **h** The degradation of CD44 and CTTN was weakened in M14-shRNA128 cells compared with that in M14-shNC cells, as demonstrated by CHX chase assays. Cells were exposed to CHX and collected at the indicated times. Cell lysates were immunoblotted with the indicated antibodies. **i** Invasion and colony formation assays were performed in the indicated cells (left); western blot was used to detect the expression of RNF128, CD44, CTTN, EMT markers (E-cadherin, vimentin, and Snail), and stemness markers (CD133) in the indicated cells (right). **j** Invasion and colony formation assays were performed in the indicated cells (left); western blot was used to detect the expression levels of RNF128, CD44, CTTN, EMT markers (E-cadherin, vimentin, and Snail), and stemness markers (CD133) in the indicated cells (right). n.s., not significant, ^**^p < 0.01, ^***^p < 0.001, ^****^p < 0.001

To elucidate the underlying mechanisms by which RNF128 exerts its function, a combination of Co-IP and MS was performed to define the interactome of RNF128. We identified 188, 172, and 149 of RNF128 interacting proteins in M14-RNF128, A2058-RNF128, and 293 T-RNF128 cells, respectively, and 10 proteins overlapped, including CD44, CTTN, Filamin-A, and Gelsolin (Fig. 4a, b). Here, we focused on CD44 and CTTN due to their high abundance in the interactome and their known oncogenic properties. RNF128 indeed formed a complex with CD44 and CTTN, as verified by Co-IP and immunofluorescence (Fig. 4c and e, Additional file 6: Figure S4A and B). Moreover, we found that RNF128 inhibition led to significantly upregulated CD44 and CTTN expression, whereas CD44 and CTTN interference did not influence the expression of RNF128 at the protein level (Fig. 4d, f), suggesting that CD44 and CTTN protein were substrates of RNF128. Unexpectedly, we found that RNF128 inhibition obviously increased the mRNA level of CD44, which indicated that RNF128 also affected CD44 expression at the transcriptional level. Next, ubiquitination assays showed that the downregulation of RNF128 inhibited CD44 and CTTN protein polyubiquitination and increased the half-life of the CD44 and CTTN proteins (Fig. 4g, h). These results suggest that RNF128 ubiquitinates and degrades CD44 and CTTN.

### Low level of RNF128 promotes melanoma cell EMT and stemness via Wnt signaling by simultaneously protecting CD44 and CTTN from degradation

To further detect the roles of CD44 and CTTN in RNF128-induced tumor progression, we interfered CD44 or CTTN expression in M14-shRNF128 cells to determine the changes in cellular EMT and stemness. We found that M14-shRNF128 cells transfected with siCD44 inhibited cell invasion and clone-forming ability, while there was no difference in M14-shRNF128 cells transfected with siCTTN (Fig. 4i, left). Western blot assays showed that M14-shRNF128 cells transfected with siCD44 exhibited a weak inhibition of shRNF128-induced EMT and stemness, which were detected by changes in EMT and stemness markers, while M14-shRNF128 cells transfected with siCTTN showed no changes in the expression of EMT and stemness markers (Fig. 4i, right). Furthermore, we transfected both siCD44 and siCTTN into M14-shRNF128 cells, and shRNF128-induced EMT and stemness were significantly attenuated (Fig. 4j). These results indicate that CD44 and CTTN synergetically promote cellular EMT and stemness induced by shRNF128.

**Fig. 5:**
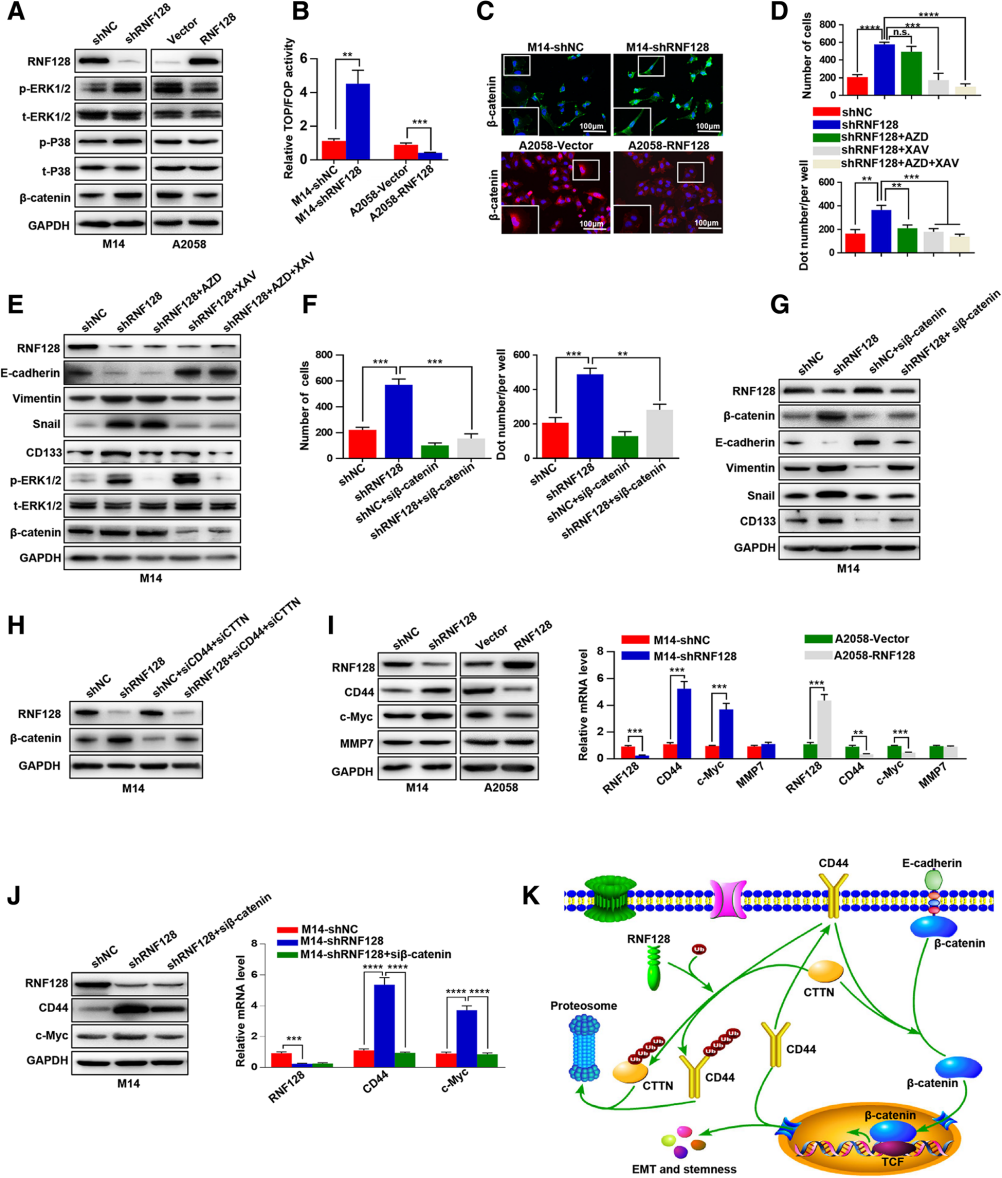
Downregulated RNF128 promotes melanoma cell EMT via positively regulating the Wnt/β-catenin-CD44 loop. **a** Western blot was used to detect the expression of MAPK and Wnt signaling-related molecules in M14-shNC/M14-shRNF128 and A2058-Vector/A2058-RNF128 cells. **b** The TOP/FOP luciferase reporter assay was performed in M14-shNC/M14-shRNF128 and A2058-Vector/A2058-RNF128 cells, and the results showed that low level of RNF128 activated the canonical Wnt/β-catenin pathway. **c** Immunofluorescence staining was used to detect the subcellular localization of β-catenin in M14-shNC/M14-shRNF128 and A2058-Vector/A2058-RNF128 cells, and the results showed that low level of RNF128 promoted the nuclear transposition of β-catenin. **d** Invasion and colony formation assays were performed in the indicated cells after incubation with inhibitors of the ERK (AZD) and Wnt (XAV) pathways, and the results showed that Wnt pathway derived low level of RNF128-induced cell invasion and stemness. **e** Western blot was used to detect the expression of RNF128, EMT markers (E-cadherin, vimentin, and Snail), CD133, p-ERK, ERK, and β-catenin in the indicated cells, and the results showed that Wnt pathway derived low level of RNF128-induced cell EMT and stemness. **f** Invasion and colony formation assays were performed in the indicated cells, and the results showed that low level of RNF128 induced cell invasion and stemness via β-catenin. **g** Western blot was used to detect the expression levels of RNF128, β-catenin, EMT markers, and CD133, and the results showed that low level of RNF128 induced cell EMT and stemness via β-catenin. **h** Western blot was used to detect the expression of RNF128 and β-catenin in the indicated cells, and it showed that low level of RNF128-induced Wnt pathway activation was decreased by CD44 and CTTN knockdown. **i** Western blot (left) and qRT-PCR (right) were used to detect the expression of target genes of Wnt/β-catenin signaling, and the results showed that low level of RNF128 promotes the expression of CD44 and c-Myc. **j** Western blot (left) and qRT-PCR (right) were used to detect the expression of CD44 and c-Myc in the indicated cells, and the results showed that downregulated RNF128 induced CD44 and c-Myc expression via Wnt/β-catenin signaling. **h** A schematic model of the positive feedback of RNF128-Wnt signaling-CD44 axis during melanoma progression.^**^p < 0.01, ^***^p < 0.001, ^****^p < 0.0001

To further explore the mechanism of RNF128-induced EMT and stemness, we analyzed the Gene Set Enrichment Analysis (GSEA) of the RNA-seq profiles of the melanoma cohort from the TCGA database and found that CD44 and CTTN levels were positively correlated with Wnt- and MAPK-activated gene signatures, suggesting that the Wnt and MAPK pathways may be involved in the function of RNF128/CD44/CTTN complex-mediated melanoma progression (Additional file 7: Figure S5). The western blot, TOPFlash/FOPFlash reporter assay, and immunofluorescence assays showed that MAPK/ERK1/2 and Wnt/β-catenin signaling were activated by low level of RNF128 (Fig. 5a–c Additional file 8: Figure S6). To examine which signaling plays critical roles in shRNF128-induced cell EMT and stemness, we treated M14-shRNF128 cells with the MEK inhibitor AZD6244 (5 μmol/L, 24 h) and/or the Wnt inhibitor XAV939 (0.6 nM, 24 h). As presented in Fig. 5d, e, XAV939 prominently inhibited cell invasion and clone-forming ability of M14-shRNF128 cells and caused corresponding changes in E-cadherin, vimentin, Snail, and CD133 expression, while AZD6244 did not influence the expression of EMT markers. Then siβ-catenin was transfected into M14-shRNF128 cells, and we found that shRNF128-induced cell invasion, clone-forming ability, EMT, and stemness were significantly reduced (Fig. 5f, g). Additionally, we transfected siCD44 and siCTTN together into M14-shRNF128 cells, and the expression of β-catenin induced by shRNF128 was attenuated (Fig. 5h). These findings indicate that shRNF128 induces EMT and stemness via Wnt/β-catenin signaling by simultaneously protecting CD44 and CTTN from degradation.

### Downregulation of RNF128 is a powerful promoter of melanoma progression via positively regulating the CD44-Wnt/β-catenin loop

Previous studies reported that CD44 [21], c-Myc [22], and MMP7 [23] are target genes of Wnt signaling, so we tested the hypothesis that low level of RNF128 promotes the positive feedback loop of Wnt/β-catenin-CD44 signaling. We found that RNF128 downregulation increased the expression of CD44 and c-Myc both in mRNA and protein levels, and vice versa, while the expression of MMP7 was not influenced by RNF128 (Fig. 5i). Furthermore, M14-shRNF128 cells transfected with siβ-catenin significantly attenuated shRNF128-induced c-Myc expression at both the mRNA and protein levels, but this treatment only slightly reduced the CD44 expression at the protein level for downregulated RNF128 protected CD44 from degradation by ubiquitination (Fig. 5j). Therefore, positive feedback occurred, and CD44, which is an accepted stemness marker, played a key role in this process. Taken together, these results indicate that RNF128 participates in the positive feedback for the Wnt signaling-CD44 axis and promotes cell EMT and stemness in melanoma cells (Fig. 5k).

### Low levels of RNF128 are associated with poor prognosis of melanoma patients

**Fig. 6:**
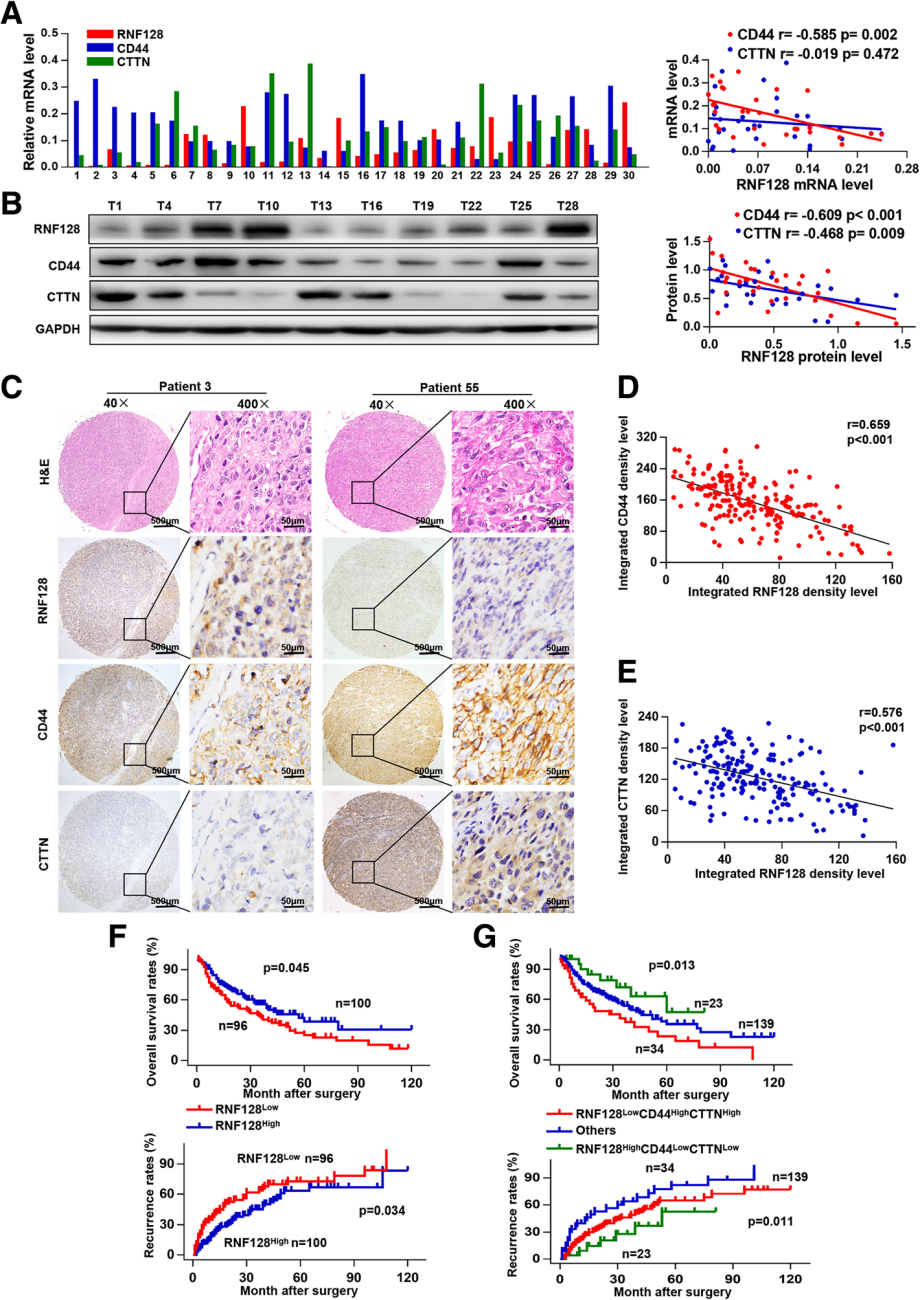
Low levels of RNF128 coupled with overexpression of CD44 and CTTN are associated with poorer prognosis in melanoma patients. **a** RNF128, CD44, and CTTN mRNA levels in 30 melanoma tissues (left), and the correlation analysis between RNF128 and CD44 or CTTN at the mRNA levels was performed using the Spearman correlation coefficient (right). **b** Representative bands of RNF128 protein levels in 30 melanoma tissues are shown (left), and the correlation analysis between RNF128 and CD44 or CTTN at the protein levels was performed using the Spearman correlation coefficient (right). **c** Representative images of TMA stained with H&E and IHC for the RNF128, CD44, and CTTN antibodies. **d**, **e** Correlation analysis of integrated RNF128 density levels with integrated CD44 (**d**) or CTTN (**e**) density levels using the Spearman correlation coefficient. **f** Kaplan-Meier analyses showing the relationship between RNF128 expression and overall survival (upper) or recurrence (lower), *p* values were calculated by the log-rank test. **g** Kaplan-Meier analyses showing the relationships between the differential expression of RNF128, CD44, CTTN, and overall survival or recurrence using the log-rank test. ^*^p < 0.05, ^***^p < 0.001. T, tumor; P, peritumor

To further explore the relationships between RNF128, CD44, and CTTN, we detected their expression in 30 pairs of melanoma and matched peritumoral tissues. RNF128 expression was negatively correlated with CD44, without correlation between RNF128 and CTTN in mRNA level (Fig. 6a). However, at the protein level, RNF128 was negatively correlated with CD44 and CTTN (Fig. 6b). Next, IHC analysis further validated the negative correlation between RNF128 and CD44 (CTTN) expression (Fig. 6c–e), which is consistent with the abovementioned results.

By prognostic analysis, we found that patients in the RNF128^Low^ group had lower overall survival rates (OS) than that in the RNF128^High^ group (*p* = 0.045, Fig. 6f, upper). The 1- and 2-year OS were 78% and 66% for the RNF128^High^ group, and only 67.7% and 51.7% for the RNF128^Low^ group respectively. Moreover, patients who scored as RNF128^Low^ had significantly higher recurrence rates than patients scored as RNF128^High^ (*p* = 0.034, Fig. 6f, lower). Strikingly, patients expressing a low level of RNF128 combined with high levels of CD44 and CTTN showed the worst prognosis (Fig. 6g). The correlation analysis demonstrated that melanoma patients with RNF128^Low^ had advanced Breslow depth, Clark level, distant metastasis, and clinical stage, as shown in Table 1. The results of univariate and multivariate analyses are shown in Table 2. Univariate analysis showed that Breslow thickness, Clark level, lymphatic metastasis, distant metastasis, clinical stage, and RNF128 staining were associated with OS and recurrence rates. Multivariate analysis showed that RNF128 represents an independent predictor for postoperative OS and recurrence rates in melanoma patients. In conclusion, low expression of RNF128 is a risk marker for OS and recurrence in melanoma patients.

**Table 1:** Correlations between RNF128 and clinicopathological features in 196 melanoma patients

Variable	Number of patients		*p* value*
	RNF128^Low^	RNF128^High^	
Age, years			
< 60	34	44	0.220
≥ 60	62	56	
Gender			
Male	49	55	0.579
Female	47	45	
Anatomic site			
Acra	56	45	0.155
Trunk	19	29	
Other	21	26	
Histologic type			
Superficial spreading	26	20	0.274
Nodular	15	26	
Acral	36	33	
Lentigo maligna	19	21	
Ulceration			
Present	15	11	0.340
Absent	81	89	
Breslow depth (mm)			
≤ 2	47	65	0.023
> 2	49	35	
Clark level			
I–III	45	61	0.047
IV–V	51	39	
Lymph nodes metastasis			
No	66	75	0.330
Yes	30	25	
Distant metastasis			
No	64	83	0.008
Yes	32	17	
Clinical stage			
I–II	50	73	0.002
III–IV	46	27	

Note: A chi-square test was used for comparing groups between low and high RNF128 expression. *p < 0.05 was considered significant

**Table 2:** Univariate and multivariate analyses of factors associated with OS and recurrence rate

Variable	OS				Recurrence			
	Multivariate analysis				Multivariate analysis			
	Univariate *p*	HR	95%CI	p*	Univariate *p*	HR	95%CI	*p*
Age, years (≥ 60 vs. <60)	0.226			NA	0.281			NA
Gender (men vs. women)	0.735			NA	0.787			NA
Anatomic site (acra vs. trunk vs. other)	0.867			NA	0.892			NA
Histologic type (superficial spreading vs. nodular vs. acral vs. Lentigo maligna)	0.095			NA	0.076			NA
Ulceration (present vs. absent)	0.267			NA	0.245			NA
Breslow depth(mm) (≤ 2 vs. > 2)	0.018*			NS	0.016*			NS
Clark level (I–III vs. IV–V)	0.015*	1.629	1.102-2.347	0.011	0.025	1.534	1.012–2.316	0.018
Lymph nodes metastasis (yes vs. no)	0.016*			NS	0.014*			NS
Distant metastasis (yes vs. no)	0.002*			NS	0.002*			NS
Clinical stage (yes vs. no)	< 0.001	3.936	2.365–6.521	< 0.001	< 0.001	3.998	2.447–6.607	< 0.001
RNF128 staining (low vs. high)	0.004	1.724	1.237–2.587	0.026	0.008	1.753	1.154–2.508	0.039

Note: *OS* overall survival, *NS* not significant, *NA* not adopt

*p < 0.05 was regarded as statistically significant, *p* value was calculated using Cox proportional hazards regression

## Discussion

The prognosis of melanoma patients remains unsatisfactory [2]. In the current study, we found that RNF128 expression was significantly downregulated in melanoma compared with that in matched peritumorous tissues, and the downregulation of RNF128 is strongly correlated with poor prognosis in melanoma patients. Moreover, we further demonstrated that low levels of RNF128 activated canonical Wnt signaling and participated in a positive feedback for the CD44-Wnt loop; thus, RNF128 functions as a tumor suppressor gene in melanoma.

RNF128 is a type I transmembrane ubiquitin-protein enzyme that localizes to the endocytic pathway [24], which has previously been reported to associate with innate and adaptive immune responses. For example, Song et al. demonstrated that RNF128 promotes IRF3 activation, IFN-β production, and innate antiviral immune responses to RNA and DNA viruses [25], and Roza et al. found that RNF128 regulation is critical for naïve T cell tolerance and regulatory T cell function, as evidenced by the greatly increased susceptibility to autoimmune diseases in Rnf128^−^/^−^ mice [26]. Recently, RNF128 was confirmed to play important roles in tumorigenesis and progression. Chen et al. found that RNF128, as a tumor promoter, physically interacts with and degrades p53 under stress conditions [27]. Conversely, Lee et al. found that downregulation of RNF128 was associated with the reduced survival in patients with urothelial carcinoma [28]. Here, we found that RNF128 expression is downregulated in melanoma compared with that in adjacent peritumoral tissues. Low levels of RNF128 were shown to induce melanoma cell EMT and promote lung metastasis through the Wnt/β-catenin pathway via the ubiquitination of CD44 and CTTN. We provide compelling evidence for the role of RNF128 in tumorigenesis and progression. Many factors may lead to the low expression of RNF128, such as methylation, miRNA regulation, and so on; further study of the upstream regulatory mechanism of RNF128 will undoubtedly be helpful to the treatment of melanoma. What is more, a better understanding of the downstream signaling pathway of RNF128 will also contribute to the therapy of melanoma.

As an E3, RNF128 physically interacts with multiple target proteins for ubiquitination and proteasomal degradation. For example, RNF128 was reported to interact with TBK1 and catalyze the K63-linked polyubiquitination of TBK1, which led to IRF3 activation and IFN-β production [25]. In our study, a combination of Co-IP and MS identified 10 proteins that might be substrates of RNF128. Among these substrates, multiple cytoskeletal regulatory proteins were found, which indicated that RNF128 plays a crucial role in cytoskeletal reorganization. These results were consistent with the previous results [29]. However, some known partners of RNF128 in lymphocytes were not found in these tumor cell lines, possibly because RNF128 plays a different role in cancer cells. Here, we focused on CD44 and CTTN due to their high abundance in the interactome and their known oncogenic properties [30, 31]. From the GEPIA analysis, the mRNA levels of these factors were slightly downregulated in tumor tissues compared with those in normal tissues (Additional file 9: Figure S7), which indicated that posttranslational modification plays crucial roles in CD44 and CTTN expression. Furthermore, when RNF128 was combined with CD44 or CTTN, RNF128-induced EMT was not evident, but when RNF128 was combined with both factors, RNF128 significantly promoted melanoma cell EMT, which indicated that these proteins play a synergistic role in activating downstream signaling pathways in melanoma cells.

EMT has been reported to be induced by multiple pathways, such as the Wnt, MAPK, and PI3K pathways [32–34]. We found that CD44 and CTTN levels were positively correlated with the Wnt and MAPK pathways, as indicated by GSEA. By western blot, we found that the RNF128/CD44/CTTN complex could activate the Wnt and MAPK pathways. Using inhibitors of Wnt and ERK1/2, we found that inhibition of Wnt signaling powerfully repressed the EMT phenotype. It has been demonstrated that activation of the Wnt pathway can induce EMT in multiple tumors, including melanoma [35–37]. Moreover, we showed that the expression of target molecules of Wnt signaling, such as CD44 and c-Myc, was regulated by RNF128 at both the mRNA and protein levels. Additionally, this upregulation of CD44 and CTTN activated the Wnt pathway and further upregulated the expression of CD44, which is a widely accepted marker in stem tumor cells [38]. Interestingly, we found that a low level of RNF128 promoted stemness in melanoma cells, which was consistent with the results of the colony formation and sphere formation assays. Thus, downregulation of RNF128 activates Wnt signaling via CD44 and CTTN ubiquitination and is involved in a positive feedback of Wnt signaling-CD44 loop.

In conclusion, we provide a reliable molecule that can be used as a potential diagnostic biomarker, prognostic indicator, and even contribute to the treatment of melanoma.

## Additional files

Additional file 1: **Table S2.** List of primary antibodies used in the study (DOCX 14 kb)

Additional file 2: **Table S1**. Sequences of primer for real-time polymerase chain reaction (DOCX 14 kb)

Additional file 3: **Figure S1.** Venn diagram showing the number of differentially expressed RNF genes in GSE3189 and GSE7553, and the names of overlapping genes are shown. (PNG 40 kb)

Additional file 4: **Figure S2.** Western blot (left) and qRT-PCR (right) analyses to detect endogenous RNF128 expression levels in six human melanoma cell lines. (PNG 61 kb)

Additional file 5: **Figure S3.** **A**, The effects of RNF128 inhibition and overexpression on apoptosis-related proteins were detected by western blot. **B**, The proliferation abilities were detected by CCK-8 assays in the indicated cells. ^***^p < 0.001, ^****^p < 0.0001. (PNG 216 kb)

Additional file 6: **Figure S4**. **A**, Histogram was used to present the colocalization of RNF128 and CD44. **B**, Histogram was used to present the colocalization of RNF128 and CTTN. ^*^p < 0.05. (PNG 65 kb)

Additional file 7: **Figure S5.** GSEA analyses showing that CD44 expression positively correlates with MAPK and Wnt signaling and that CTTN expression positively correlates with MAPK signaling. (PNG 825 kb)

Additional file 8: **Figure S6.** A histogram was used to present the expression of MAPK and Wnt signaling-related molecules by western blot using Student’s *t* test. ^**^p < 0.01, ^***^p < 0.001, ^****^p < 0.0001. (PNG 41 kb)

Additional file 9: **Figure S7.** The mRNA levels of CD44 and CTTN in melanoma tissues compared with those in normal tissues in the TCGA database analyzed by GEPIA. The cutoff was |Log_2_FC| ≥ 1 and *p* value< 0.01. (PNG 60 kb)

## Abbreviations

CCK-8: Cell Counting Kit-8
CTTN: Cortactin
DAPI: 4, 6-Diamidino-2-phenylindole
E3s: Ubiquitin-protein enzymes
EMT: Epithelial-mesenchymal transition
GEO: Gene Expression Omnibus
GSEA: Gene Set Enrichment Analysis
H&E: Hematoxylin and eosin
HA: Hemagglutinin
IHC: Immunohistochemistry
IP: Immunoprecipitation
MMP9: Matrix metalloproteinase-9
MS: Mass spectrometry
qRT-PCR: Quantitative real-time polymerase chain reaction
RNFs: Ring finger proteins
SD: Standard deviation
TMA: Tissue microarray
