# Supplementary material for: Downregulation of RNF128 activates Wnt/β-catenin signaling to induce cellular EMT and stemness via CD44 and CTTN ubiquitination in melanoma
Source: J Hematol Oncol. 2019 Mar 4;12:21. doi: 10.1186/s13045-019-0711-z (PMC6399928; doi:10.1186/s13045-019-0711-z)
Supplement: Supplementary file 1 — Table S2. List of primary antibodies used in the study (DOCX 14 kb) [file 13045_2019_711_MOESM1_ESM.docx]

**Supplementary Table II List of Primary Antibodies Used In the study**

| Antibody | Applications | Company |
| --- | --- | --- |
| RNF128 | WB, IHC, IF | Abcam (ab72533) |
| GAPDH | WB, IF, IHC | Abcam (ab8245) |
| E-cadherin | WB, F, IF,IHC | CST (3195) |
| Vimentin | WB, F, IF,IHC | CST (5741) |
| Snail | WB, IP, IF | CST (3879) |
| CD133 | WB, IHC,IF | CST (64326) |
| Twist | WB, IHC, IF | Abcam (ab50581) |
| α-tubulin | WB, IHC, IF, F | Abcam (ab18251) |
| Lamin B1 | WB, IP, IHC | Abcam (ab133741) |
| CD44 | WB, IP, IHC, IF, F | CST (5640) |
| CTTN | WB, IF, IP | CST (3503) |
| HA | WB, IP, IF, ELISA | Proteintech (51064-2-AP) |
| V5 | WB, IP, ELISA | Proteintech (66007-1-lg) |
| Flag | WB, IP, IF | Proteintech (66008-2-lg) |
| p-ERK | WB, IP, IHC,IF, F | CST (4370) |
| ERK | WB, IP, IHC, IF,F | CST (4695) |
| p-P38 | WB, IP, IHC, IF,F | CST (4511) |
| P38 | WB, IHC, IF,F | CST (8690) |
| p-JNK | WB, IP, IF, F | CST (9255) |
| JNK | WB, IP, IHC, F | Abcam (ab208035) |
| p-β-catenin | WB, IF, ELISA | Abcam (ab27798) |
| β-catenin | WB, IP, IHC, IF, F, ChIP | CST (8480) |
| c-Myc | WB, IP, IHC, F | Abcam (ab32072) |
| MMP7 | WB, IHC | CST (3801) |

**Abbreviations:** WB, western blot; IHC, immunohistochemistry; IF, immunofluorescence; IP, immunoprecipitation; ELISA, enzyme linked immunosorbent assay; F, Flow cytometric analysis; ChIP, Chromatin immunoprecipitations.
