# Supplementary material for: Downregulation of RNF128 activates Wnt/β-catenin signaling to induce cellular EMT and stemness via CD44 and CTTN ubiquitination in melanoma
Source: J Hematol Oncol. 2019 Mar 4;12:21. doi: 10.1186/s13045-019-0711-z (PMC6399928; doi:10.1186/s13045-019-0711-z)
Supplement: Supplementary file 2 — Table S1. Sequences of primer for real-time polymerase chain reaction (DOCX 14 kb) [file 13045_2019_711_MOESM2_ESM.docx]

**Supplementary Table I Sequences of Primer for Real-time Polymerase Chain Reaction**

| **RNF128** |  |
| --- | --- |
| Forward | 5’-TGGCCCTGATGGAGATAGTTG-3’ |
| Reverse | 5’-GCACGTTAAGATGCGTACCAA-3’ |
| **E-cadherin** |  |
| Forward | 5’-ATTTTTCCCTCGACACCCGAT-3’ |
| Reverse | 5’-TCCCAGGCGTAGACCAAGA-3’ |
| **Vimentin** |  |
| Forward | 5’-AGTCCACTGAGTACCGGAGAC-3’ |
| Reverse | 5’-CATTTCACGCATCTGGCGTTC-3’ |
| **Snail** |  |
| Forward | 5’-TCGGAAGCCTAACTACAGCGA-3’ |
| Reverse | 5’-TCGGAAGCCTAACTACAGCGA-3’ |
| **CD133** |  |
| Forward | 5’-AGTCGGAAACTGGCAGATAGC-3’ |
| Reverse | 5’-GGTAGTGTTGTACTGGGCCAAT-3’ |
| **CD44** |  |
| Forward | 5’-CTGCCGCTTTGCAGGTGTA-3’ |
| Reverse | 5’-CATTGTGGGCAAGGTGCTATT-3’ |
| **CTTN** |  |
| Forward | 5’-GTGGTTTTGGCGGCAAGTATG-3’ |
| Reverse | 5’-CTCTCTGTGACTCGTGCTTCT-3’ |
| **c-Myc** |  |
| Forward | 5’-GTCAAGAGGCGAACACACAAC-3’ |
| Reverse | 5’-TTGGACGGACAGGATGTATGC-3’ |
| **MMP7** |  |
| Forward | 5’-GAGTGAGCTACAGTGGGAACA-3’ |
| Reverse | 5’-CTATGACGCGGGAGTTTAACAT-3’ |
| **GAPDH** |  |
| Forward | 5’-GGTATGACAACGAATTTGGC-3’ |
| Reverse | 5’-GAGCACAGGGTACTTTATTG-3’ |
