# Supplementary figures and images for: Downregulation of RNF128 activates Wnt/β-catenin signaling to induce cellular EMT and stemness via CD44 and CTTN ubiquitination in melanoma
Source: J Hematol Oncol. 2019 Mar 4;12:21. doi: 10.1186/s13045-019-0711-z (PMC6399928; doi:10.1186/s13045-019-0711-z)

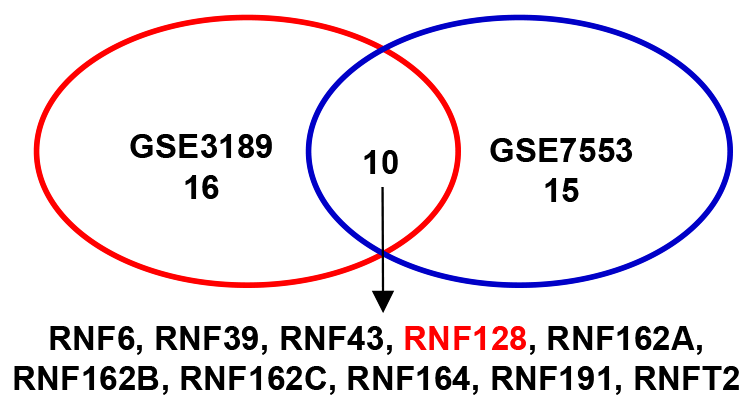

Supplement: Supplementary file 3 — Figure S1. Venn diagram showing the number of differentially expressed RNF genes in GSE3189 and GSE7553, and the names of overlapping genes are shown. (PNG 40 kb) [file 13045_2019_711_MOESM3_ESM.png]

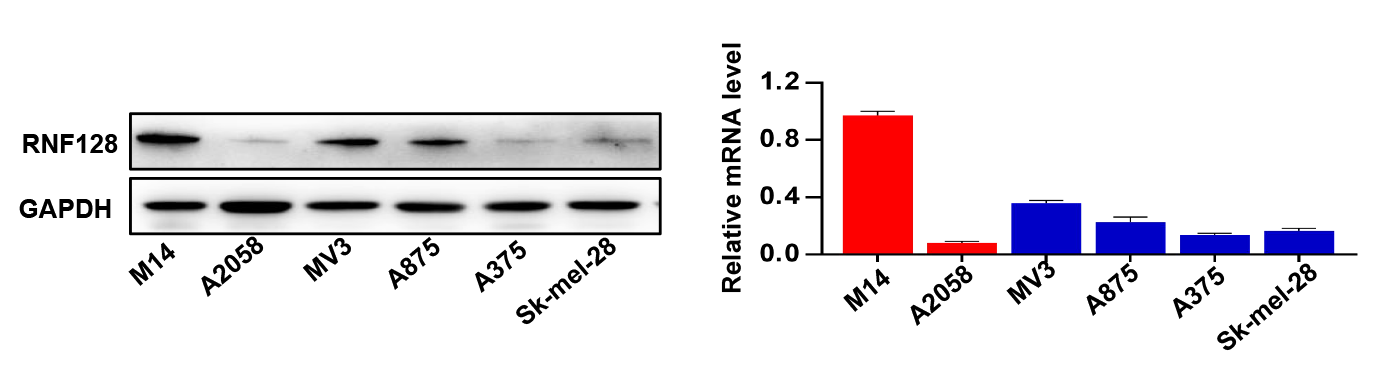

Supplement: Supplementary file 4 — Figure S2. Western blot (left) and qRT-PCR (right) analyses to detect endogenous RNF128 expression levels in six human melanoma cell lines. (PNG 61 kb) [file 13045_2019_711_MOESM4_ESM.png]

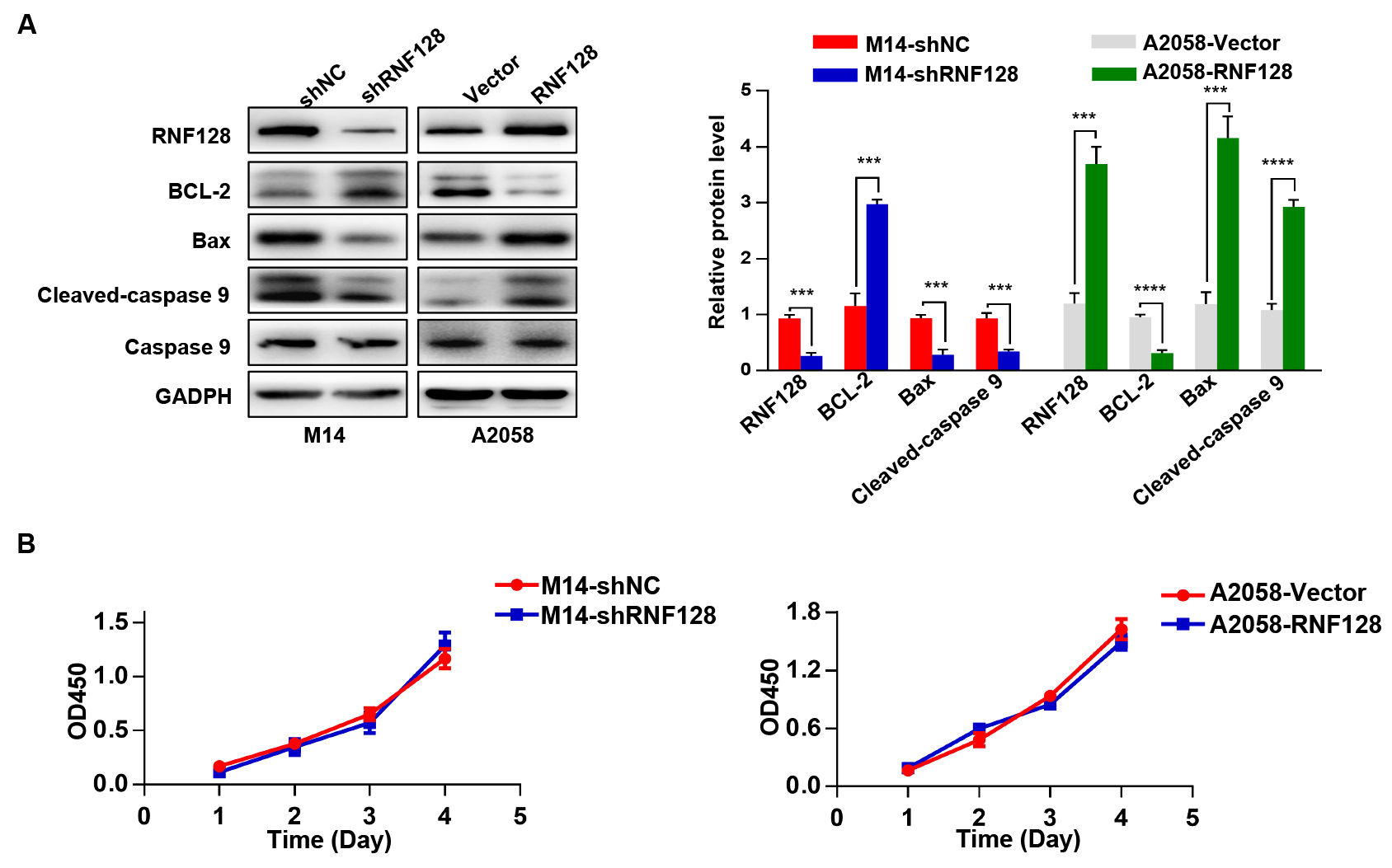

Supplement: Supplementary file 5 — Figure S3. A, The effects of RNF128 inhibition and overexpression on apoptosis-related proteins were detected by western blot. B, The proliferation abilities were detected by CCK-8 assays in the indicated cells. ***p < 0.001, ****p < 0.0001. (PNG 216 kb) [file 13045_2019_711_MOESM5_ESM.png]

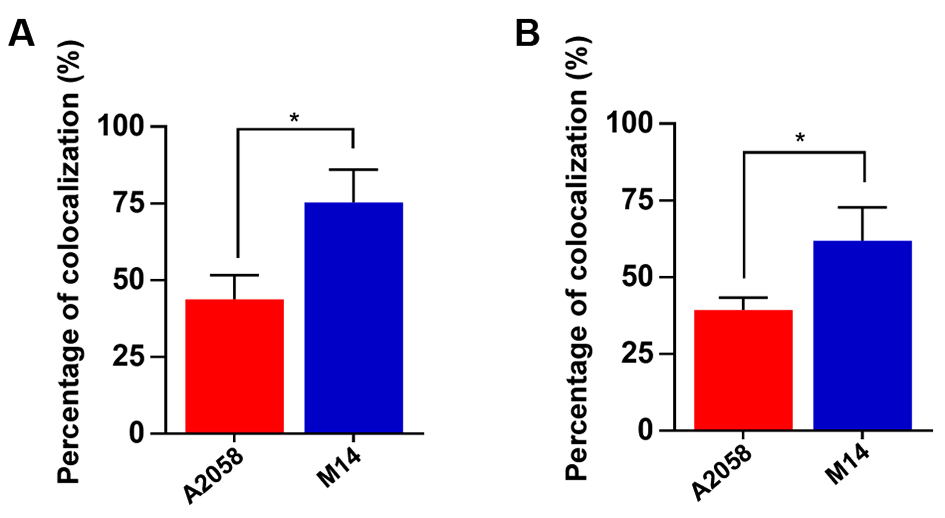

Supplement: Supplementary file 6 — Figure S4. A, Histogram was used to present the colocalization of RNF128 and CD44. B, Histogram was used to present the colocalization of RNF128 and CTTN. *p < 0.05. (PNG 65 kb) [file 13045_2019_711_MOESM6_ESM.png]

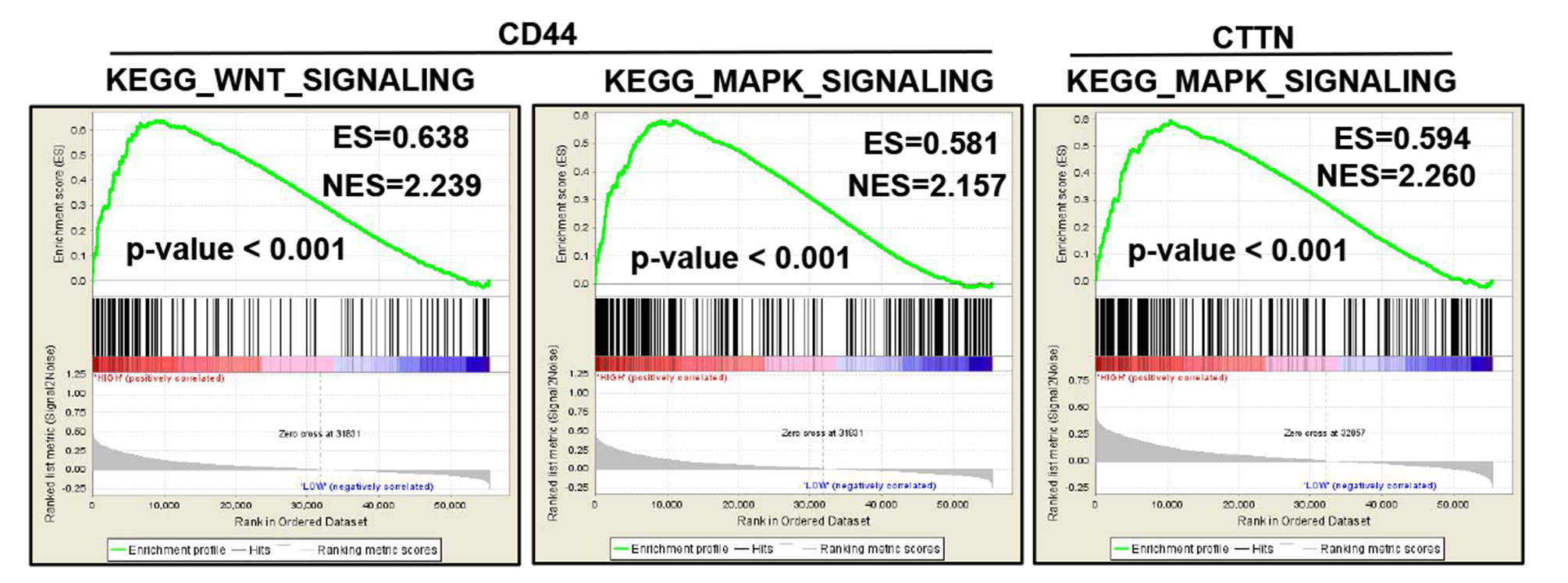

Supplement: Supplementary file 7 — Figure S5. GSEA analyses showing that CD44 expression positively correlates with MAPK and Wnt signaling and that CTTN expression positively correlates with MAPK signaling. (PNG 825 kb) [file 13045_2019_711_MOESM7_ESM.png]

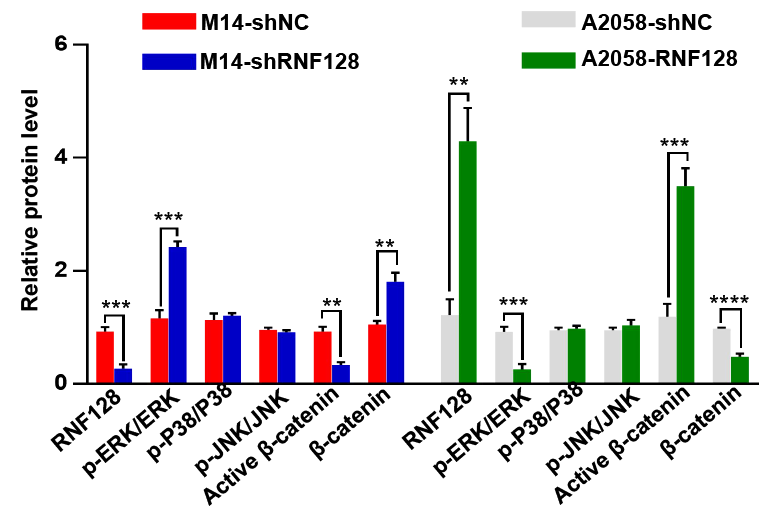

Supplement: Supplementary file 8 — Figure S6. A histogram was used to present the expression of MAPK and Wnt signaling-related molecules by western blot using Student’s t test. **p < 0.01, ***p < 0.001, ****p < 0.0001. (PNG 41 kb) [file 13045_2019_711_MOESM8_ESM.png]

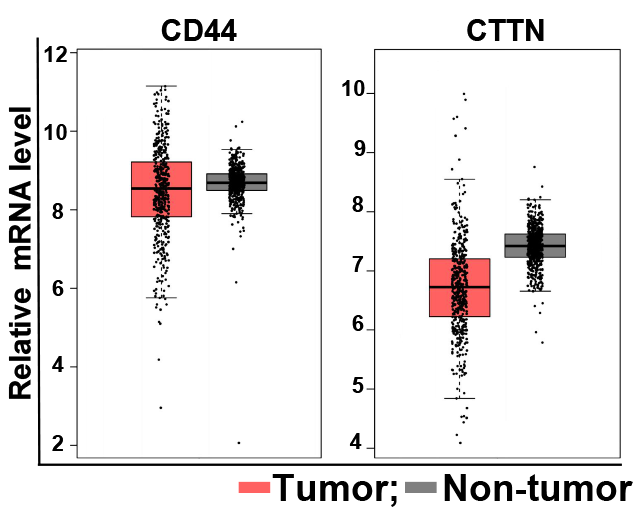

Supplement: Supplementary file 9 — Figure S7. The mRNA levels of CD44 and CTTN in melanoma tissues compared with those in normal tissues in the TCGA database analyzed by GEPIA. The cutoff was |Log2FC| ≥ 1 and p value< 0.01. (PNG 60 kb) [file 13045_2019_711_MOESM9_ESM.png]
